# Quality evaluation of commercially available male condoms in Rio de Janeiro, Brazil, 2009–2011

**DOI:** 10.1186/s12978-016-0254-5

**Published:** 2016-12-07

**Authors:** Janete Teixeira Duarte, Antonio Eugenio C. C. de Almeida, Shirley de Mello Pereira Abrantes

**Affiliations:** 1 National Institute for Quality Control of Health - INCQS, Oswaldo Cruz Foundation - FIOCRUZ, Av. Brasil, 4365, Rio de Janeiro, CEP: 21040-900 RJ Brazil; 2 Departamento de Química - INCQS/Fiocruz, Oswaldo Cruz Foundation/National Institute of Health Quality Control (INCQS), Fundação Oswaldo Cruz/Instituto Nacional de Controle de Qualidade em Saúde, Av. Brasil, 4365, Rio de Janeiro, CEP > 21040-360 Brazil

**Keywords:** Male Condoms, Public Health, Sanitary Surveillance

## Abstract

**Background:**

The increased incidence of sexually transmitted infections (STIs) in Brazil represents a significant public health issue. This issue has raised awareness among health authorities regarding the quality of condoms. In Brazil, male condoms need to be certified. The certification process evaluates in detail the manufacturing and quality of the final product; however, post-market surveillance is not part of the normal certification practice.

**Methods:**

From 2009 to 2011, we evaluated 20 male condoms brands per lot of 8 manufactures-both domestic and imported-marketed in Rio de Janeiro, Brazil. Sampling was performed per ISO 2859–1, and the condoms were evaluated on length, width, thickness, holes, integrity of primary packaging, bursting volume, bursting pressure, label and secondary packaging, following the criteria established in the Brazilian National Health Oversight Agency Resolution no. RDC 62/2008.

**Results:**

Of the 20 evaluated brands, 17 brands were found to be noncompliant with the guidelines of the Brazilian National Health Oversight Agency Resolution no. RDC 62/2008 in at least one of the analyses performed.

**Conclusions:**

Any nonconforming unit has serious public health implications.

## Background

The quality of condoms available in Brazil has been widely debated since 1987, when condoms were included in the category of pharmaceuticals. Condoms remain under the jurisdiction of the Brazilian Sanitary Surveillance Agency (*Agência Nacional de Vigilância Sanitária*: ANVISA) under the Brazilian Ministry of Health. The first Brazilian law, Brazilian National Health Oversight Agency Resolution no. 3 in 2002, repealed by RDC 62/2008, was based on the ISO 4074:2002 standards that established the technical regulation for certification of male condoms of natural rubber latex; it specified that condoms must be certified under the Brazilian Certification System in accordance with the requirements of the indicated technical regulations before their sale or free distribution to consumers.

**Fig. 1:**
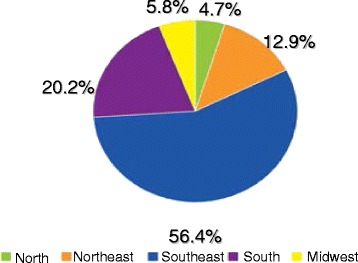
Percentage distribution of AIDS cases by region of residence. Brazil, 1980–2011

Male condoms serve a dual function by protecting against both pregnancy and sexually transmitted infections (STIs) [1–4]. The incidence of STIs has increased in Brazil and now represents a significant public health issue. This increase is predominantly due to low socioeconomic status, poor cultural conditions, and lack of adequate sex education, particularly among young individuals. Today, STIs are among the most common diseases worldwide [5, 6]. For example, of the 656.701 cases of AIDS in Brazil identified from 1980 to June 2012, 61.400 (9.3%) were reported per the case definition for death criteria consisting of 41.459 (67.5%) males and 19.933 (32.5%) females [6]. Figure 1 shows percentage distribution of AIDS cases by region of residence (Brazil, 1980–2011).

A critical analysis of the Brazilian response to fight HIV/AIDS emphasized the importance of integrating prevention, care, and treatment under the Unified Health System [Sistema Único de Saúde (SUS)] with reference to human rights [7].

Globally, the health impact of STIs has led to the allocation of substantial funds for treatment of young individuals in a range of countries. The World Health Organization (WHO) estimated the rise in incidence of curable STIs in Brazil from 10 million to 12 million annually, covering the age group of 15–49 years [8].

In Brazil, the use of condoms remains an important prevention policy. The Ministry of Health, in response to the increase in cases of STIs and the consideration of the potential consequences, evaluated inconsistencies in the quality of condoms and the disinterest in the use of condoms throughout the population. To remedy this, postexposure prophylaxis has been introduced for the treatment of all high-risk individuals following sexual intercourse (vaginal, anal and oral) since October 2010. This involves the administration of medications up to 72 h after sexual intercourse if condoms were not used or had failed during intercourse [9].

The quality of latex condoms may vary according to the lot, differences in manufacturing technology and the batch of manufacture, resulting in considerable quality variation. From the point of view of contraception, the condom would fail on the order of 15 to 100 on the Pearl Index, i.e., 15 pregnancies in 100 women who use the method each year [10].

The tests established in RDC 62/2008 for the evaluation of condoms include the measurement of dimensional properties (length, width, and thickness), bursting volume, and bursting pressure, in addition to inspection for the detection of holes or other defects. Labeling accuracy and packaging integrity were additionally evaluated. Sampling procedures were performed according to the ISO 2859–1 sampling plans for inspection by attributes through the lot size, the number of units to be evaluated, the acceptance criteria, and the acceptable quality level of acceptable qualities for each type of test, in addition to inspection levels [11].

The male condom is a simple, inexpensive, and widely used material used to prevent unwanted pregnancy and transmission of STIs, but it requires sophisticated oversight by governmental institutions to ensure quality. In this regard, the actions of sanitary surveillance post-marketing have become essential in monitoring condoms for reducing potential public health risks [12].

The objective of the present study was to evaluate the physical quality of condoms marketed in Rio de Janeiro, Brazil.

## Methods

One out of 600 units from each brand was collected for the post-marketing surveillance, and we evaluated 20 domestic and imported condom brands per lot from eight manufacturers marketed in Rio de Janeiro. The condoms were anonymized by marking them with the letters A through H corresponding to each manufacturer and the numbers 1 through 20 for each brand. Condoms were tested according to the standards established by the Brazilian National Health Oversight Agency Resolution no. RDC 62/2008 [11]. Table 1 shows the number of samples necessary to carry out each test, the acceptable quality level from each test and the criteria established in RDC 62 for the quality control testing of condoms. The ISO 2859–1 sampling procedures for inspection by attributes define the acceptance criteria, the level of inspection (I, II, or III) and the inspection regime based on the size of the lot [13].

**Table 1:** Criteria established in RDC 62 for the quality control testing of condoms

PHYSICAL TEST	AQL	Number of samples tested	Maximum acceptable non-conforming units
Length, width, thickness	4.0	13	2
Holes	0.25	315	3
Primary packaging integrity	2.5	20	2
Bursting volume and bursting pressure	1.5	200	8
Label and secondary packaging	1.0	13	1

*AQL* Acceptable quality level

Evaluated dimensional properties include condom length, width and thickness, which generally range from 160 to 200, 45 to 60 and 0.03 to 0.09 mm, respectively. Condom width defines condom size and is primarily determined by the circumference (twice the flat width) [11].

Of all the tests performed, the absence of holes is considered the most important and can be performed using an electrical or visual method. The electrical method involves the filling of suspended condoms with an electrolytic aqueous solution of sodium hydroxide, and then the passage of an electric current is used to indicate the presence of holes. The visual method involves filling suspended condoms with water and detecting leaks. Both methods provide equivalent results [14]. Both tests were utilized for evaluating the samples [15].

The bursting volume must be ≥ 16.0 dm^3^ for condoms with a width ≤ 50.0 mm; ≥ 18.0 dm^3^ for condoms with a width > 50.0 and ≥ 22.0 dm^3^ for condoms with a width ≥ 56.0. The bursting pressure must be ≥ 1 kPa regardless of the width [11]. Bursting volumes and pressure were measured using an eight-head automated inflation system (Enersol, Sydney, Australia). Condoms were inflated with the latex film stretched until rupture as a measure of maximum resistance. The inflation system is accompanied by software [16] that records the bursting volumes and pressure. The compressed air that supplies the system is generated by an oil-free air compressor. The flow of compressed air was set at 24–30 dm^3^ min^−1^ for all evaluations, as defined in the standards. For each condom, the bursting pressure and bursting volume were recorded with EInflation3 software [15] (Enersol’s head office is in Sydney Australia). For label and packaging inspection, we evaluated all items according to Appendix A of RDC 62/2008 [11].

## Results and discussion

Twenty different condoms brands, 13 domestic and 7 foreign, were analyzed. Table 2 shows the results for each test performed for each of the 20 condoms. We identified defective samples in 17 lots in one or more assay, with the results summarized in Table 3.

**Table 2:** Test results for the twenty condom brands

ANALYZED SAMPLE	Dimensions (length, width, thickness)	Freedom from holes	Package integrity	Volumetric capacity and burst pressure	Packaging and labeling
A1					
D2				NC	
C3		NC			
A4			NC		
F5		NC		NC	
G6				NC	
H7				NC	
H8	NC				
B9		NC		NC	
D10					
D11				NC	
B12				NC	
E13				NC	
E14					NC
D15				NC	
A16				NC	
A17				NC	
A18				NC	
A19				NC	
D20					

*NC* Not complying

**Table 3:** Number of defective samples found by tests in the twenty brands analyzed

Dimensions (length, width, thickness)	1
Freedom from holes	3
Package integrity	1
Volumetric capacity and burst pressure	13
Packaging and labeling	1

According to RDC 62/2008, condom width is required to be within 2 mm of the declared nominal width. All brands analyzed had the stated nominal width of 52 mm.

**Fig. 2:**
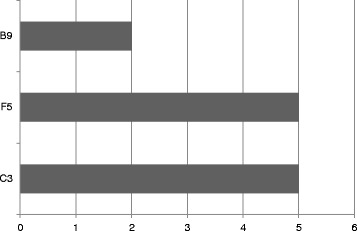
Results of the freedom from holes test

The freedom from holes test evaluated 315 units of each brand according to the sampling plan of ISO 2859–1. The requirements of RDC 62 for assessment of lots specify an AQL of 0.25 and a maximum of two nonconforming units. The amount of holes analysis is the parameter treated with the greatest rigor in all standard tests. The present analysis found that three brands had units that were noncompliant: two were beyond the limits established by law (C3 and F5). Figure 2 shows the results obtained in the holes verification analysis in male condoms.

The results show that the B9 brand, although it complies with the criteria of RDC 62/2008, provided two non-conforming units, representing a health risk.

The package integrity analysis predominantly assesses primary sealing of condoms; according to RDC 62/2008, the packaging must be hermetically sealed and opaque to protect the product from contact with oxygen, ozone, moisture, and visible and ultraviolet light that degrade natural rubber. The packaging material must be laminated metallic material, flexible, and waterproof, with inner layers made of plastic (polyethylene). While evaluating package integrity, we found a nonconforming unit (A4) considering the criteria of the Board Resolution (RDC 62). However, the Sanitary Surveillance, in addition to the criteria established by the legislation, shows that non-compliance can lead to product contamination, which may cause damage to the health of users.

**Fig. 3:**
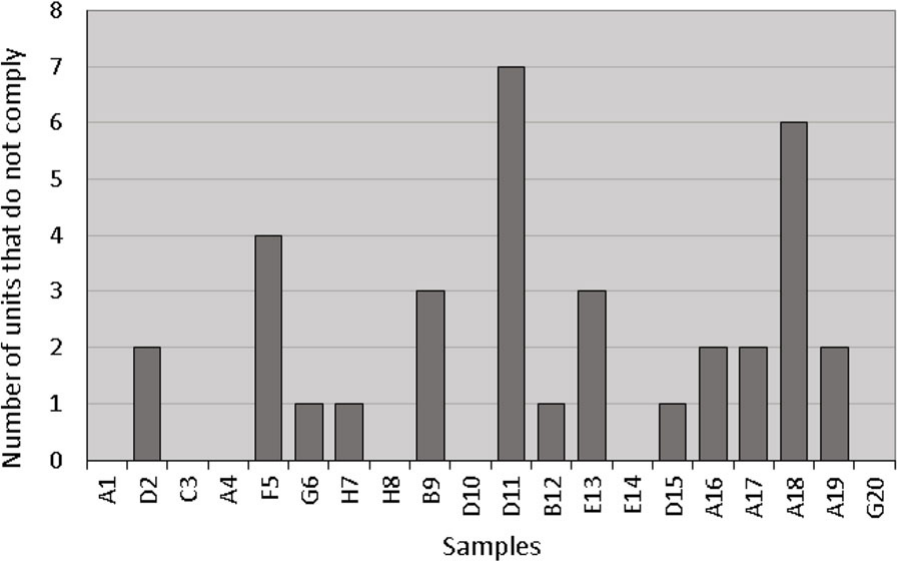
Numbers of noncompliant samples from the analysis of volumetric capacity and burst pressure

The main causes of condom failure during use are rupture and slipping. The main advantage of the analysis of volumetric capacity and overflow, other than assessing failure rates, is the ability to assess the entire condom, sensitivity to faults located in the film, and a possible correlation between low test performance degradation and condom aging in the present study [17]. In total, 200 units of each of the 20 brands were evaluated, and 17 nonconforming units were identified, indicating a greater need for the monitoring of currently marketed condoms to reduce the imminent public health risk. Figure 3 shows the numbers of noncompliant samples.

## Conclusion

We observed noncompliance in at least one of the analyses performed on the 17 brands. The bursting volume and bursting pressure tests identified 13 noncompliant brands, with three noncompliant brands identified during the holes check analysis. These results demonstrate that the main objectives of the use of condoms in preventing STIs and protecting against pregnancy are not guaranteed.

The result of this study showed two shortfalls of the criteria laid down in legislation. However, any non-compliance from the point of view of surveillance, even within the legally established limits, can pose serious public health implications.
